# Effects of Fish Predators on the Mass-Related Energetics of a Keystone Freshwater Crustacean

**DOI:** 10.3390/biology9030040

**Published:** 2020-02-25

**Authors:** Douglas S. Glazier, Jonathan J. Borrelli, Casandra L. Hoffman

**Affiliations:** 1 Department of Biology, Juniata College, Huntingdon, PA 16652, USA; 2 Department of Biological Sciences, Rensselaer Polytechnic Institute, Troy, NY 12180, USA; borrej4@rpi.edu; 3 Department of Pediatrics, School of Medicine, University of Virginia, Charlottesville, VI 22908, USA; hoffmcl08@gmail.com

**Keywords:** body-mass scaling, fat content, fish predation, food assimilation, freshwater springs, *Gammarus minus*, growth rate, metabolic rate, ontogeny, reproduction

## Abstract

Little is known about how predators or their cues affect the acquisition and allocation of energy throughout the ontogeny of prey organisms. To address this question, we have been comparing the ontogenetic body-mass scaling of various traits related to energy intake and use between populations of a keystone amphipod crustacean inhabiting freshwater springs, with versus without fish predators. In this progress report, we analyze new and previously reported data to develop a synthetic picture of how the presence/absence of fish predators affects the scaling of food assimilation, fat content, metabolism, growth and reproduction in populations of *Gammarus minus* located in central Pennsylvania (USA). Our analysis reveals two major clusters of ‘symmorphic allometry’ (parallel scaling relationships) for traits related to somatic versus reproductive investment. In the presence of fish predators, the scaling exponents for somatic traits tend to decrease, whereas those for reproductive traits tend to increase. This divergence of scaling exponents reflects an intensified trade-off between somatic and reproductive investments resulting from low adult survival in the face of size-selective predation. Our results indicate the value of an integrated view of the ontogenetic size-specific energetics of organisms and its response to both top-down (predation) and bottom-up (resource supply) effects.

## 1. Introduction

The life and death struggle between predators and prey is one of the most striking ecological interactions in nature. Predators not only kill their prey (a consumptive effect), but also intimidate them (a non-consumptive effect), both of which may alter their biology and ecology in many ways [1,2,3,4,5]. Prey responses may involve long-term, genotypic, adaptive evolution in populations (e.g., [6,7,8]) or short-term, phenotypically plastic acclimation by individuals (e.g., [2,5,9]). Predators may induce changes in a wide variety of morphological, physiological, behavioral and life-history traits of their prey (e.g., [1,2,5,7,9,10,11,12]). However, relatively little is known about how predators affect the way that prey organisms expend energy for various vital activities during their ontogeny [13,14]. A complete understanding of predator effects on prey should embrace all of the life stages, especially since predation is often age- and size-specific (e.g., [6,7,8,13,15,16,17,18,19,20,21,22]). Furthermore, centrally important prey responses include changes in rates of foraging, locomotor activity, growth and reproduction that ultimately relate to energy intake and use [5,11,14]. Previous studies have indicated that predation risk may cause increases, decreases or no changes in the metabolic rate of prey organisms, and these varied responses may even occur within the same species (Table 1). However, the effect that age or body size of prey has on metabolic responses to predators is largely unknown [13,23,24].

Therefore, the purpose of this article is to present a progress report on an ongoing project focused on how fish predators affect the ontogenetic body-mass scaling of energy intake and expenditure in the freshwater amphipod *Gammarus minus*. Toward this end, we compare new data on mass-specific food assimilation rate (with associated fat content of individual amphipods in the laboratory) and re-analyzed, previously published data on mass-related resting metabolic rate, gill surface area, growth, reproduction and field-based fat content between populations inhabiting freshwater springs with versus without fish predators. 

Although further data are needed, especially in the field, our analyses so far suggest that the body-mass scaling of energy expenditure in organisms need not be entirely driven by resource-supply constraints, as often assumed (e.g., according to the metabolic theory of ecology, MTE [25,26]), but may also be influenced by resource demand (cf. [13,27,28,29,30]). Moreover, contrary to the MTE, metabolic scaling may not be the simple result of bottom-up effects of resource supply, but may also be affected by top-down effects of predators, or interactions between these effects. Our results indicate that fish predators have caused a suite of traits related to somatic investment to shift their allometric scaling in a congruent way, but in an opposite way to that of traits related to reproductive investment, a pattern that can be explained by life-history theory (see Section 3.6 and Section 4.3). As such, they indicate the insightful value of taking an integrated view of how predators affect the ontogenetic scaling of energy intake and expenditure in prey organisms. Our results also have implications for theoretical growth models based on comparing the scaling of assimilation rate with that of the rate of maintenance or resting metabolism (see Section 4.5). In addition, they highlight the importance of adaptive evolution and biological regulation in the body-mass scaling of energetic processes and its response to ecological factors.

**Table 1:** Positive (POS), negative (NEG) or no (NO) effects of predators or their cues on the resting or routine metabolic rate ^1^ of various prey species.

Predator	Prey Species	Metabolic Response	Source
**AQUATIC**			
Octopus	*Jasus edwardsii* (decapod)	NEG ^2^	[31]
Octopus	*Gobius paganellus* (fish)	NEG ^2^	[32]
Seastar	*Fissurella limbata* (mollusk)	POS ^2^	[33]
Odonate larvae	*Ischnura elegans* (odonate)	NO ^2,3^	[34]
Odonate larvae	*Libellula quadrimaculata* (odonate)	NO ^2,3^	[34]
Odonate larvae	*Sympetrum sanguineum* (odonate)	NO ^2,3^	[34]
Odonate larvae	*Rana temporaria* (tadpole)	POS/NEG ^2,3,4^	[23]
Odonate larvae	*Bufo arabicus* (tadpole)	NEG/NO ^2,3,4^	[35]
Fish	*Gammarus minus* (amphipod)	POS/NEG ^5,6^	[13]
Fish	*Daphnia magna* (cladoceran)	NO ^2^	[36]
Fish	*D. magna* (cladoceran)	POS ^2^	[37]
Fish	*D. magna* (cladoceran)	NO ^2^	[38]
Fish	*D. magna* (cladoceran)	NEG ^2^	[39]
Fish	*Panopeus herbstii* (decapod)	NO ^2^	[40]
Fish	*Dreissena polymorpha* (mollusk)	NEG/NO/POS ^2,4^	[41]
Fish	*Enallagma cyathigerum* (odonate)	POS ^2^	[42]
Fish	*E. vesperum* (odonate)	POS ^2^	[43]
Fish	*Carassius auratus* (fish)	NEG ^2,3^	[44]
Fish	*C. carassius* (fish)	NEG ^2^	[45]
Fish	*Fundulus majalis* (fish)	POS ^2^	[46]
Fish	*Galaxias maculatus* (fish)	NEG/NO ^2,7^	[47]
Fish	*Parabramis pekinensis* (fish)	POS ^2^	[24]
Fish	*Pimephales promelas* (fish)	POS ^2^	[39]
Fish	*Poecilia reticulata* (fish)	NEG ^6^	[48]
Fish	*P. reticulata* (fish)	NEG/NO ^2,8^	[48]
Fish	*P. reticulata* (fish)	POS ^6^	[49]
Fish	*Pseudorasbora parva* (fish)	POS ^2^	[50]
Fish	*Spinibarbus sinensis* (fish)	POS ^2,3^	[51]
Fish	*Zacco platypus* (fish)	POS ^6^	[52]
**TERRESTRIAL**			
Spider	*Hasarius adansoni* (spider)	POS/NEG ^2,4^	[53]
Spider	*Melanoplus fenurrubrum* (grasshopper)	POS ^2^	[54]
Mantid insect	*Argiope keyserlingi* (spider)	POS ^2^	[55]
Pentatomid insect	*Leptinotarsa decemlineata* (beetle)	POS/NEG/NO ^2,8^	[56,57]
Pentatomid insect	*Manduca sexta* (caterpillar)	POS ^2,3^	[58]
Reptile	*Teleiogryllus commodus* (cricket)	POS ^2^	[14]
Rat	*Tenebrio molitor* (beetle)	NEG ^6^	[59,60]
Bird	*Parus major* (bird)	NO ^2^	[61]
Frightening stimulus	*Sylvilagus aquaticus* (mammal)	NEG ^2^	[62]
Mammal	*Cervus elaphus* (mammal)	POS ^2^	[63]
Mammal	*Erinaceus europaeus* (mammal)	POS ^2^	[64]
Mammal	*E. europaeus* (mammal)	NEG ^2^	[65]

^1^ Based on measurements of O_2_ uptake, CO_2_ release, or the doubly labelled water method. ^2^ Short-term individual responses involving phenotypically plastic acclimation. ^3^ Prey are juveniles. ^4^ Response depends on duration of exposure to predators. ^5^ Response depends on age and body size. ^6^ Long-term population responses involving differential survival or adaptive evolution. ^7^ Response depends on type of predator cue. ^8^ Response depends on other interacting environmental factors.

## 2. Materials and Methods

### 2.1. Study System

Our study system consists of *Gammarus minus* populations inhabiting freshwater springs with and without the predatory fish *Cottus cognatus*. *G. minus* is an omnivorous scavenger that commonly occurs in groundwater, springs and headwater streams with a limestone geology in the mid-Appalachians west to the Ozarks in North America [66,67]. The six study populations occur in small lotic (rheocrene) springs located in Huntingdon County of south-central Pennsylvania, USA. These springs are physically and chemically similar, all having high water clarity, shallow depth (<0.5 m), moderate water velocity (~0.1–1 m/s), a heterogeneous substrate (including cobbles, gravel, sand and silt) with patches of macrophytes (mosses and/or watercress), and nearly constant water temperatures (9–12 °C) and ionic composition (relatively alkaline with nearly neutral pH, ranging from 6.6 to 7.7) [13,68,69,70]. However, three of the springs (Ell, Blue and Williamsburg) contain predatory fish (*C. cognatus*), whereas three others (Petersburg, Kanesatake and Big Rock) are fishless [13,71]. 

Our study springs are useful because their similar, naturally controlled physical and chemical conditions permit us to use them as informative natural experiments [13,69,70,71,72,73,74]. They also have relatively few dominant animal and plant species, thus simplifying the interpretation of comparative analyses [68,75]. 

Amphipods are also useful to study because (1) they are readily collected and studied in the laboratory, (2) their body-mass range is large (ranging over an order of magnitude), thus increasing the power of scaling analyses, (3) the uniformity of their postembryonic morphology (neonates look like adults) simplifies scaling analyses, (4) they have low rates of dispersal and gene flow, thus facilitating adaptive evolutionary responses of populations to local environmental conditions, as evidenced by significant inter-population genetic differentiation [67,76,77,78] and divergence in various morphological, physiological and life-history traits [13,67,69,70,71,72,73,79], and (5) they are keystone species that play major roles in trophic energy flow and nutrient cycling in our study springs and many other aquatic ecosystems [80].

In addition, the predator *C. cognatus* is a critical component of our study system because it is a size-selective visual hunter. It prefers to eat large versus small amphipod prey [20], and this size-selective mortality has major consequences for the evolution of adult body size, rates of growth and metabolism, and other morphological, physiological, behavioral and life-history traits in *G. minus* populations that are relevant to our study [13,70,71,73,81]. 

### 2.2. Amphipod Collection

Juvenile and adult amphipods of a wide variety of sizes (~30-fold variation in dry body mass) were collected to ensure statistically adequate scaling analyses. All adults used to measure rates of assimilation, metabolism and growth were males to prevent complications in our analyses arising from eggs carried by females. Wet and (or) dry masses (± 1 μg) of individual amphipods were determined on a Cahn C-31 microbalance (Cahn Instruments, Cerritos, CA, USA). All of the energetic measurements, both in the field and laboratory, were made on amphipods in their native spring water. 

### 2.3. Measuring Food Assimilation Rate in the Laboratory

All feeding trials were carried out in an environmental control room (10 °C = average spring water temperature) during June to July 2009. Before each feeding trial, freshly collected amphipods were fasted for 24 h to allow them to void all feces resulting from food consumed in the wild (during starvation, a gammarid gut is evacuated within 24 h at temperatures ranging from 5–15 °C [82]). Each amphipod was fasted in a 250 mL plastic container separated into upper and lower chambers by horizontal 1 mm nylon mesh. As a result, feces could accumulate in the bottom chambers where the coprophagous amphipods could not eat them. 

Immediately after fasting, the food ingestion rate of each amphipod was determined over a 4-day period in a two-chambered container, as above, only this time the upper chamber contained a pre-weighed, 1 cm diameter elm (*Ulmus americana*) leaf disk as food (following [83,84]). Detrital elm leaves, an especially palatable food for freshwater amphipods [85,86,87] were collected freshly fallen during autumn 2008, air-dried and stored in plastic bags. Before use, they were soaked in tap water overnight to make them more pliable. Disks were cut using a cork borer, and then conditioned in nylon 0.3 mm mesh bags submerged in Williamsburg Spring for four weeks, an optimal time for the colonization of peak levels of microbes and fungi (see Appendix A) that enhance the palatability and nutritional value of detrital leaves to amphipods and other detritivores [85,86,87,88,89]. The leaf disks were conditioned in Williamsburg Spring because its water has a similar temperature (~11 °C) and chemical composition (pH ~ 7.4–7.8, conductivity ~ 180–260 µS) to that of the four springs that were sources for the amphipods used in our assimilation study, and using it for leaf conditioning prevented any inter-population bias in food consumption that may have resulted if the leaf conditioning occurred in one of the study springs. After conditioning, the leaf disks were dried in an oven at 50 °C and stored in desiccators until needed. 

The spring water used in the food ingestion trials was filtered with Whatman GF/C 1.2-μm glass fiber filters to remove bacteria and organic matter that may have affected the food or estimated feeding rate of the amphipods. During each feeding trial, the feeding containers (with perforated caps and external wax-covered metal weights) were submerged in 15 L plastic basins of filtered spring water to minimize changes in water chemistry during the feeding trials. After four days of incubation, the remains of the leaf disk in each feeding container were dried at 50 °C and reweighed. After correcting for the effect of leaching, the difference in dry mass of a leaf disk before and after a feeding trial indicated the amount of food ingested by an amphipod. The amount of mass lost by leaching was determined by weighing leaf disks before and after four days in feeding chambers without amphipods (three controls per 15 amphipod treatments). Energy ingestion (*I*, J day^−1^) was calculated as:

*I* = [(*L_i_* ∙ *L_c_*) − *L_f_*] *E_L_* ∙ *t*^−1^ (1)

where *L_i_* is the initial dry mass (mg) of a leaf disk; *L_c_* is a correction factor for mass loss due to leaching of soluble materials from the leaf disks; *L_f_* is the final dry mass (mg) of a leaf disk; *E_L_* is the energy equivalent of leaf dry mass (J mg^−1^): and *t* is duration of a feeding trial (= 4 d). *L_c_* was calculated as:

*L_c_* = *L_cf_*/*L_ci_* (2)

where *L_cf_* and *L_ci_* refer to final and initial dry masses of control leaves placed in containers without amphipods. Energy assimilation (*A*, J day^−1^) was calculated as:

*A* = *I* ∙ *Æ* (3)

where Æ is assimilation efficiency [(*I* − *F)I*, where *F* is feces production], which was estimated as 0.2, based upon estimates for *G. pseudolimnaeus* (0.19: [89]) and *G. minus* (0.21: unpublished data for Ell Spring amphipods carried out in the context of the energy-budget study reported by [72]), both feeding on conditioned elm leaves. At the end of each feeding trial, the leaf disks were removed from each feeding chamber and the amphipods were allowed to evacuate their guts for 24 h. Then each amphipod was stored at −70 °C, freeze-dried for 60 h, and weighed. The energy content of the elm-leaf food (*E_L_*) was determined using a Phillipson oxygen bomb calorimeter (Gentry and Wiegert Instruments, Inc., Aiken, SC, USA). *E_L_* was considered to be 18.5 J mg^−1^ based on mean estimates (± S.E.) for elm leaves conditioned in Williamsburg and Ell springs respectively [18.6 *±* 0.6 (*N* = 2); 18.1 *±* 0.6 (*N* = 8)]. 

### 2.4. Measuring Fat Content

The fat composition of the amphipods used in the feeding trails was estimated because we wished to test whether different energy-storage levels (and thus presumably different ‘hunger levels’) affected energy assimilation. This was important because individuals of *G. minus* from fish springs have significantly lower fat levels and mass per length than those from fishless springs [69,72], probably because the presence of predatory fish inhibits the foraging and energy assimilation of amphipods [13,90,91,92,93]. Fat was extracted from dried amphipods used in the food assimilation tests by placing each individual in 5 mL of diethyl ether, changed daily for five days (following [69,81,94]), and then oven-dried at 50 °C for 24 h. Fat mass was determined as dry mass minus fat-free dry mass.

We also used data on fat content of freshly collected juvenile and adult amphipods (from [69]) to calculate scaling relationships between fat mass and dry body mass in populations from Petersburg, Kanesatake, Ell and Blue springs. 

### 2.5. Estimating Scaling Exponents of Food Assimilation Rate in the Field

We inferred scaling exponents for assimilation rates in the field (*b_F_*) by using scaling relationships between fat mass and dry body mass measured in juvenile and adult male and non-brooding female amphipods freshly collected from the field to adjust scaling exponents for assimilation rate measured in the laboratory under ad libitum food conditions (*b_L_*). We assumed that relative fat mass is a reliable indicator of food availability or accessibility in the field (see also [81]), and that deviations of fat-mass scaling exponents (*b_FAT_*) from isometry (slope = 1) represented size-related deviations in accessible food supply. For example, *b_FAT_* > 1 would indicate an increase in size-specific food supply with increasing body size, whereas *b_FAT_* < 1 would indicate a decrease in size-specific food supply with increasing body size. Based on these assumptions, we estimated *b_F_* using the following formula:

*b_F_* = *b_L_* + (*b_FAT_* − 1) (4)

### 2.6. Measuring Growth Rates 

Data on growth rates were taken from [13], where the methods are described in more detail. Mass-specific growth rates were estimated for individual amphipods cultured in field enclosures for three weeks in each of their home springs during June to August 2008. Growth rates were measured for five size classes (1–2, 3–4, 5–6, 7–8, and 9–10 mm body length) by comparing final wet mass (*M*_f_) with initial wet mass (*M*_i_) (except for Ell and Blue springs, where the largest size class was absent). The field enclosures were translucent plastic cylinders (55 mm length and 25 mm diameter for 1–4 mm juveniles; 80 mm length and 55 mm diameter for 5–10 mm adults) capped at each end with 0.3-mm nylon mesh allowing water circulation with the environment. Each enclosure contained a single amphipod with sufficient natural detrital food to last three weeks. Relative growth rates (*RGR*) were estimated as:

(log_10_*M*_f_ − log_10_*M*_i_)/*t* (5)

where *t* is time (days).

### 2.7. Measuring Rates of Resting Metabolism

Data on resting metabolic rates (*RMR*) were taken from [13], where the methods are described in more detail. Metabolic (respiration) rates were estimated as oxygen consumption rates of individual amphipods using a Strathkelvin respirometry system (Strathkelvin Instruments, Glasgow, UK) in an environmental control room (10 °C). Each amphipod was fasted for 24 h (to remove the energetic costs of specific dynamic action) before being placed in a respirometer (glass syringe) containing natural spring water filtered with Whatman GF/F 0.7 µm glass fiber filters to remove bacteria and fine organic matter. Pieces of nylon mesh in each respirometer minimized amphipod movement and associated energy expenditure. Rate of oxygen consumption (*R_O2_*) over a ~6-h incubation period was estimated by comparing the oxygen content of water samples from treatment syringes, each containing an amphipod, with that of control syringes without amphipods. *R_O2_* was converted to rate of energy expenditure per day (*R_E_*) by using an oxyjoule equivalent of 21 J mL^−1^ of oxygen consumed [95].

### 2.8. Estimating Energetic Costs of Maintenance, Growth and Reproduction

The mass-specific energy cost of non-growth body maintenance (*C_M_*) was estimated by calculating the *RMR* at the dry body mass at which growth was calculated to cease, by using the empirical scaling relationships of growth and *RMR* with dry body mass. First, the growth scaling relationships were determined as wet final mass (*M_f_*) versus initial wet mass (*M_i_*) (see Section 2.6). Second, the maximal wet mass at which growth ceased was estimated at the point where *M_f_* = *M_i_*. Third, the maximal wet mass was converted to dry mass by using the empirical scaling relationships between wet and dry body masses given in Appendix B. Fourth, the *RMR* at this non-growing maximal mass (i.e., *C_M_*) was determined from empirical *RMR* scaling relationships reported in [13]. Fifth, following the theoretical growth models of [96,97,98], the body-mass scaling of *C_M_* was then calculated based on the assumption that the slope is 1. The energy cost of growth (*C_G_*) and its scaling with dry body mass was in turn estimated by comparing the scaling relationships of *RMR* and *C_M_*. In effect,

*C_G_* = *RMR* − *C_M_*. (6)

Total mass of eggs in a brood was used as a proxy for the energy cost of reproduction (reproductive investment). Dry brood mass, brood size and dry egg mass data all in relation to dry body mass from Petersburg, Kanesatake and Big Rock springs (without fish predators) and Ell and Blue springs (with fish predators) were taken from [71]. 

### 2.9. Scaling Analyses

**Figure 1:**
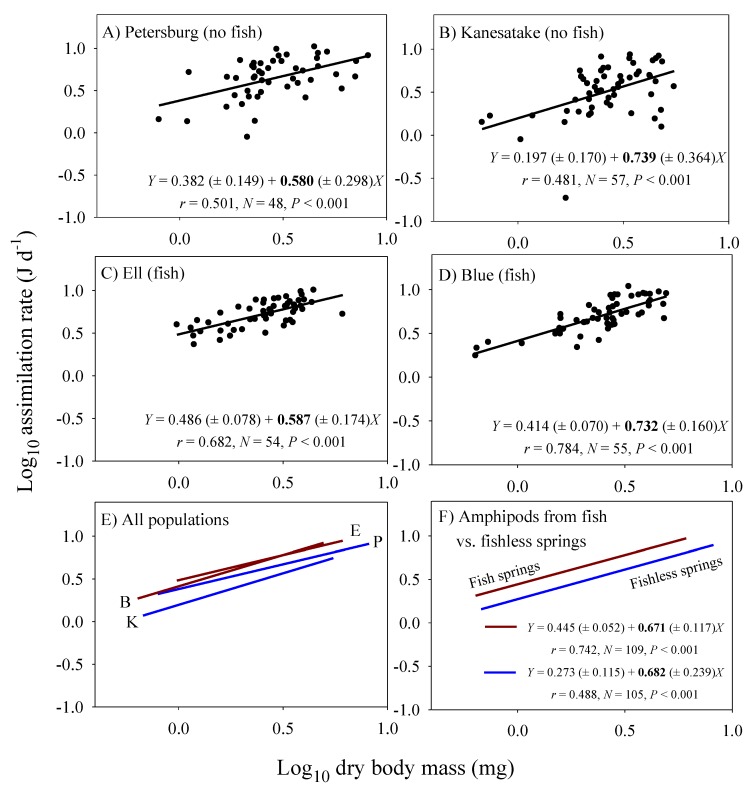
Log_10_ food-energy assimilation rate in relation to log_10_ dry body mass in four populations of the amphipod *Gammarus minus* (based on data in Supplementary Materials). Least squares regression lines and equations are shown (numbers in parentheses are 95% confidence intervals for the intercepts and slopes; *r* = correlation coefficient; *N* = sample size; *P* = probability that the correlation is due to chance). Scaling relationships for two populations in springs without fish predators (*Cottus cognatus*) (**A**,**B**), for two populations with fish predators (**C**,**D**), for all four populations compared (**E**), and for the populations with versus without fish, each analyzed collectively (**F**).

Allometric scaling relationships are commonly determined by using least squares regression (LSR) or reduced major axis (RMA) analyses [99,100,101]. Here we used LSR because it is more appropriate than RMA when the *X* variable is thought to be influencing the *Y* variable, rather than the reverse [99], and when the measurement error is substantially larger for *Y* than *X*, as appears to be the case for the rates of growth, metabolism and assimilation (*Y* variables) relative to that of body mass (*X*) (cf. [100,101]). As shown in [13] and Figure 1 in this paper, the measured rates of growth, metabolism and assimilation can vary considerably (as much as an order of magnitude) for amphipods having the same body mass. All data were log transformed to normalize the variation and to enable proportional relationships to be readily discerned (after [102,103]). The significance of differences among scaling exponents (slopes) and intercepts was estimated by ANCOVA (with body mass as a covariate), and by comparing their means and 95% confidence intervals. If a mean value was outside the 95% CI of another mean value and vice versa, they were considered significantly different (*P* < 0.05), following [104]). 

## 3. Results

### 3.1. Organization of Results

First, we present scaling analyses for food assimilation rates in the laboratory, and then use scaling analyses of fat content in wild-caught amphipods to infer the scaling exponents for food assimilation rates in the field. Second, we present scaling analyses of rates of growth and resting metabolism, and use them to estimate scaling of maintenance metabolism and the energetic cost of growth. Third, we present scaling analyses of various measures of reproductive investment, including total mass and number of eggs per brood and mean individual egg mass. Fourth, we provide an integrated description of all of the scaling relationships analyzed, and how they are influenced by fish predators. 

### 3.2. Scaling of Food Assimilation Rate in the Laboratory

Significant linear relationships between log_10_ food energy assimilation rate and log_10_ dry body mass are seen in all four population samples of *Gammarus minus* (Figure 1). Although the elevations (intercepts) of these scaling relationships are significantly higher for the populations from the fish versus fishless springs (ANCOVA: *F*_3, 209_ = 17.517; *P* < 0.001), the scaling exponents (slopes) are not significantly different among the populations (ANCOVA interaction between population and body mass: *F*_3, 206_ = 0.445; *P* = 0.721). Furthermore, the scaling exponents for all of the sampled amphipods from each of the pairs of populations from the fish (*b* = 0.671) versus fishless springs (*b* = 0.682) are similar and nearly equal to 2/3.

**Figure 2:**
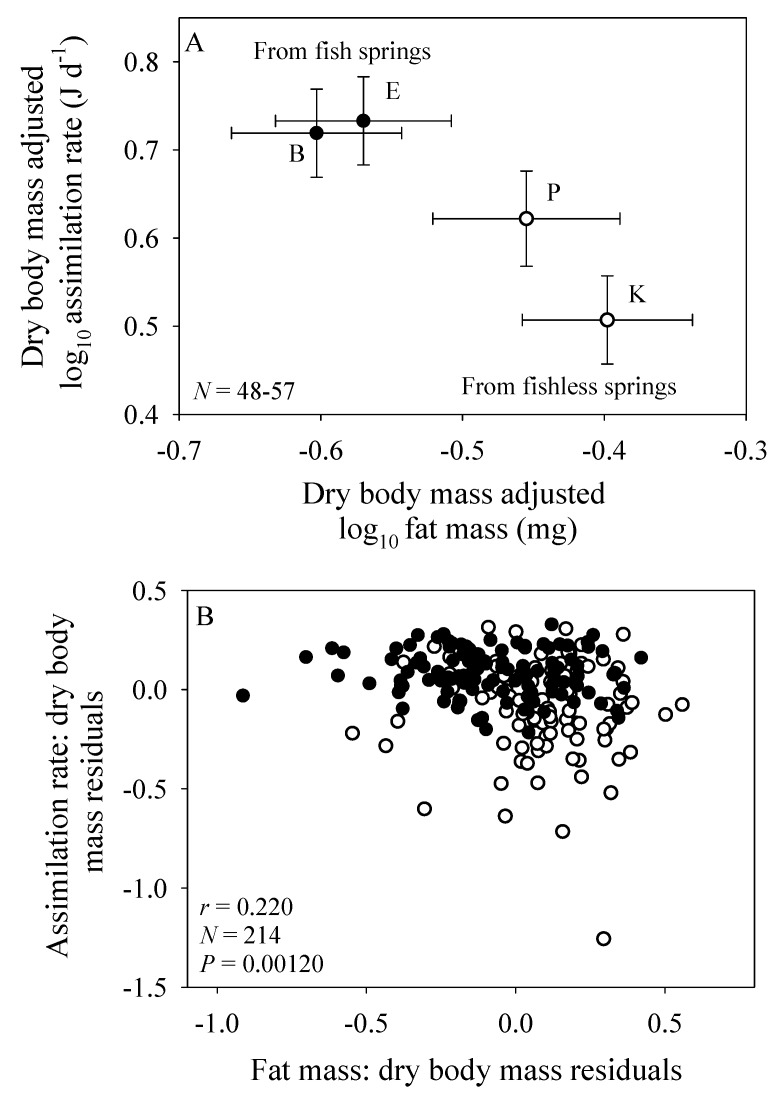
Assimilation rate as a function of fat mass in four populations of the amphipod *Gammarus minus*, two from springs with fish (solid circles) and two from springs without fish (open circles) (based on data in Supplementary Materials). (**A**) Shown are mean values (± 95% confidence intervals) adjusted to dry body mass by ANCOVA for each population and the range of sample sizes (*N*) among all four populations. (**B**) Shown are individual values expressed as residuals from least squares regressions against dry body mass for all four populations collectively. Shown are the correlation coefficient (*r*), sample size (*N*) and probability that the correlation between the residuals of assimilation rate and fat mass is due to chance (*P*).

The population samples from the springs with fish predators have both a significantly higher mean body-mass adjusted assimilation rate and a significantly lower mean body-mass adjusted fat mass than those from springs without fish (Figure 2A). Therefore, across populations assimilation rate is inversely related to body-fat content (Figure 2A). This inverse correlation is also seen among individuals from all four populations analyzed collectively using residual analysis (Figure 2B).

### 3.3. Scaling of Fat Mass of Field-Collected Amphipods, with Inferences about the Scaling of Feeding Rate in Nature 

**Figure 3:**
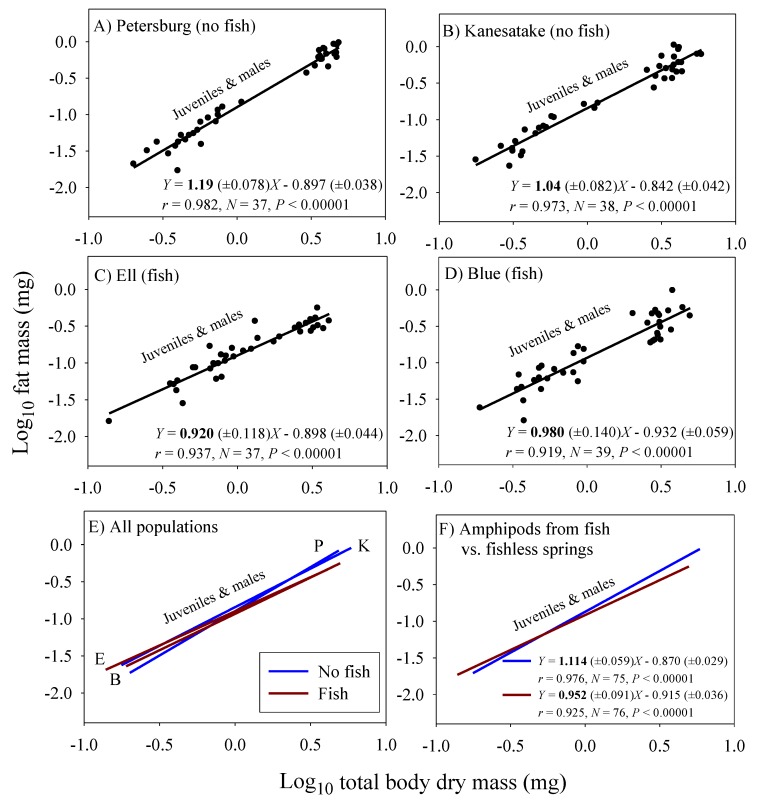
Log_10_ fat mass in relation to log_10_ dry body mass for juveniles and adult males in four populations of the amphipod *Gammarus minus*. Least squares regression lines and equations are shown (numbers in parentheses are 95% confidence intervals for the intercepts and slopes; *r* = correlation coefficient; *N* = sample size; *P* = probability that the correlation is due to chance). Scaling relationships for two populations in springs without fish predators (*Cottus cognatus*) (**A**,**B**), for two populations with fish predators (**C**,**D**), for all four populations compared (**E**), and for the populations with versus without fish, each analyzed collectively (**F**).

**Figure 4:**
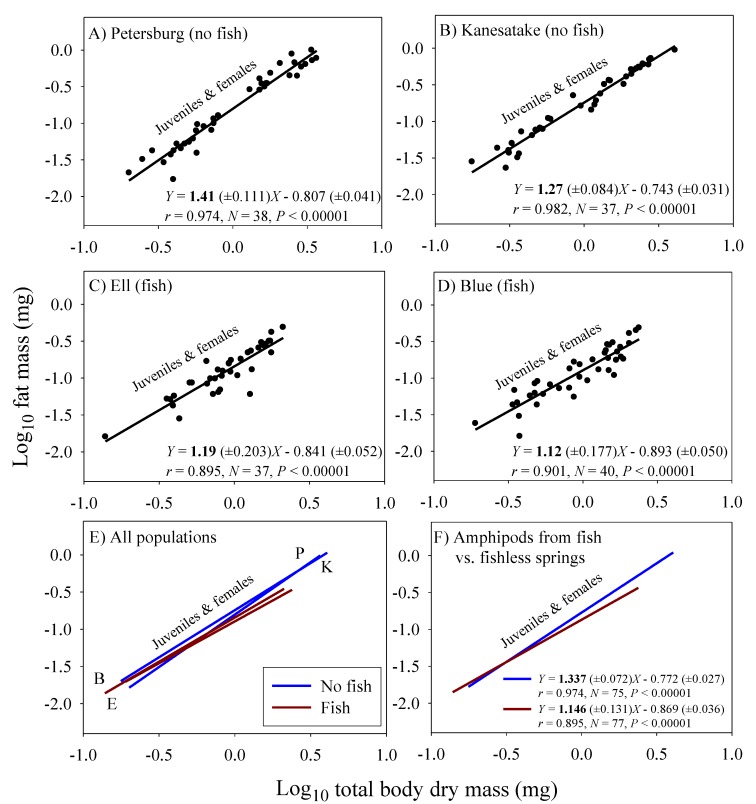
Log_10_ fat mass in relation to log_10_ dry body mass for juveniles and adult non-brooding females in four populations of the amphipod *Gammarus minus*. Least squares regression lines and equations are shown (numbers in parentheses are 95% confidence intervals for the intercepts and slopes; *r* = correlation coefficient; *N* = sample size; *P* = probability that the correlation is due to chance). Scaling relationships for two populations in springs without fish predators (*Cottus cognatus*) (**A**,**B**), for two populations with fish predators (**C**,**D**), for all four populations compared (**E**), and for the populations with versus without fish, each analyzed collectively (**F**).

**Figure 5:**
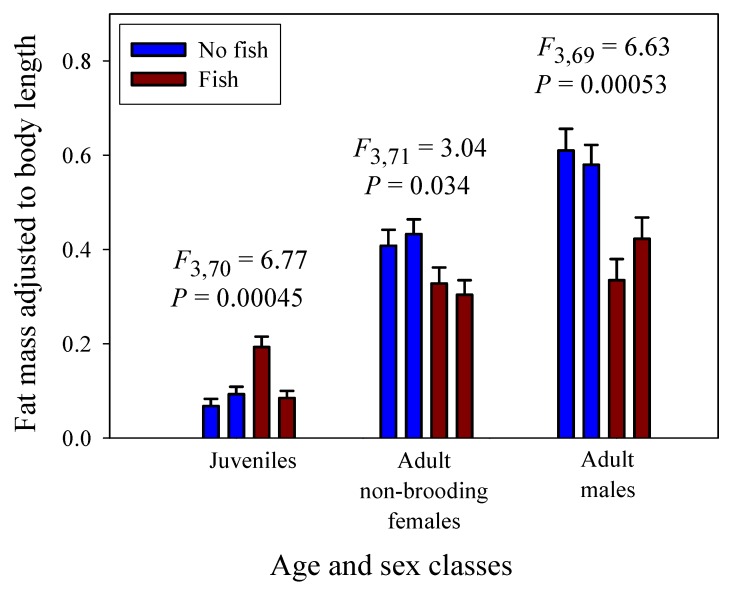
Mean fat mass (±95% confidence intervals) adjusted to body length by ANCOVA in juveniles, adult non-brooding females and adult males from four populations of the amphipod *Gammarus minus*, two from springs with fish (red bars) and two from springs without fish (blue bars). Shown are *F* and *P* values from the ANCOVA analyses for each size and sex class.

Significant linear relationships between log_10_ fat mass and log_10_ dry body mass are seen in all eight samples of *G. minus* (Figure 3 and Figure 4). For samples based on juveniles and adult males, the scaling slopes are significantly higher for the two populations from springs without fish than those for the two populations from springs with fish (Figure 3F). A similar pattern is seen for the samples based on juveniles and adult non-brooding females (Figure 4F). However, the scaling slopes tend to be higher for the samples of juveniles and non-brooding females, perhaps because of increased fat accumulation for egg production. The differences in fat-mass scaling slopes between populations with versus without fish arise from juveniles tending to show similar or significantly higher fat contents in springs with versus without fish, whereas adult males and non-brooding females show significantly lower fat contents in springs with versus without fish (Figure 5). Clearly, predation regime has a size-specific effect on body fat content. 

The fat-mass scaling exponents (*b_FAT_*) can be used to adjust the scaling exponents for assimilation rate observed in the laboratory under ad libitum food conditions (*b_L_*) to infer the scaling exponents for assimilation rate in the field (*b_F_*), by using equation 4. As a result, *b_F_* is inferred to be lower for the populations inhabiting springs with fish (0.671 − 0.048 = 0.623) than for the populations inhabiting springs without fish (0.682 + 0.114 = 0.796). 

### 3.4. Scaling of Costs of Growth and Maintenance

**Figure 6:**
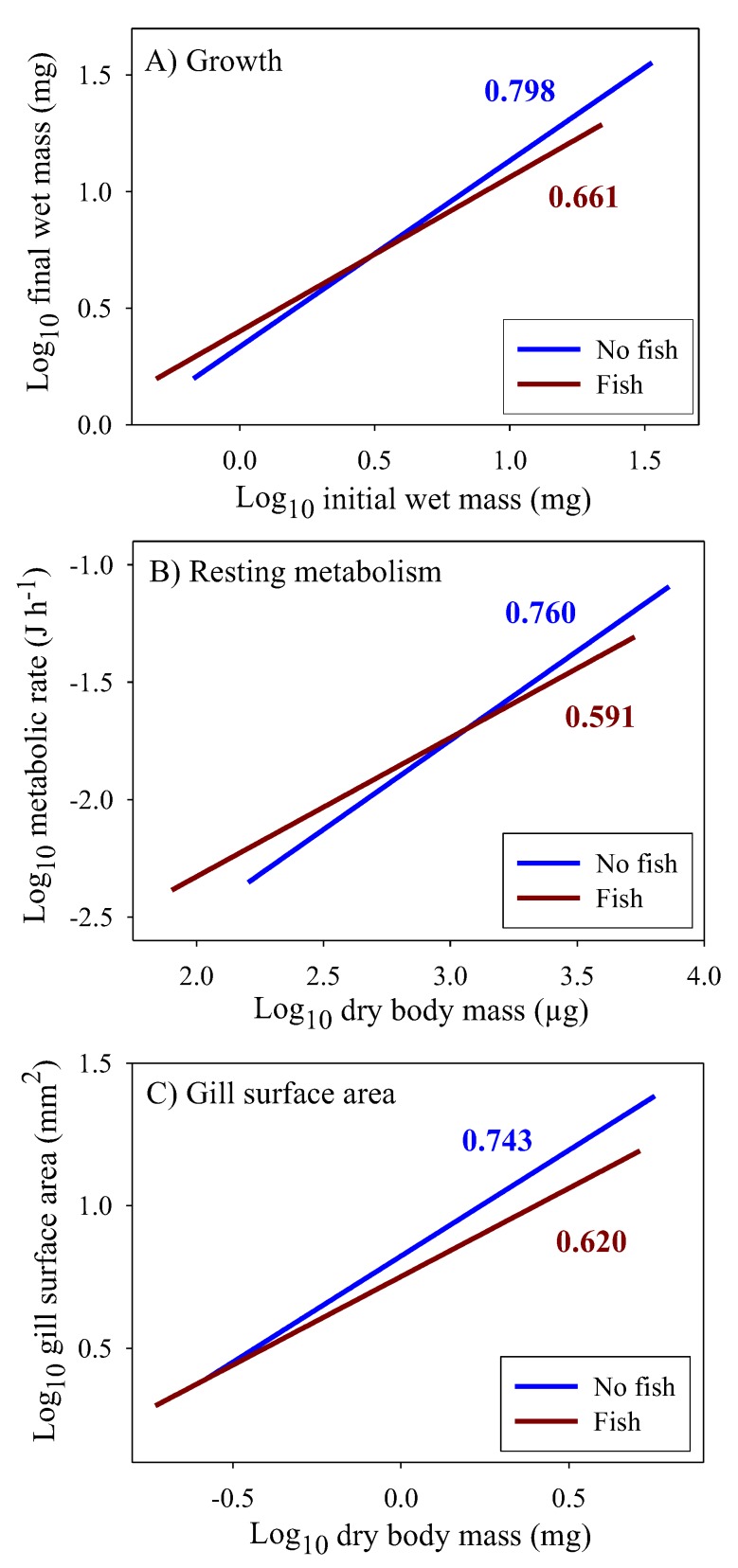
Comparisons of body-mass scaling relationships for growth rate, resting metabolic rate and gill surface area between population samples of *Gammarus minus* from springs with (*F*) versus without fish predators (*Cottus cognatus*) (*NF*). (**A**) Log_10_ final wet mass in relation to log_10_ initial wet mass (data for Petersburg, Kanesatake, Ell and Blue populations from [13]. The regression equations (including 95% confidence intervals for each parameter) are (*NF*): *Y* = 0.798 (± 0.030) *X* + 0.335 (± 0.030) (*r* = 0.961; *N* = 228; *P* < 0.00001) and (*F*): *Y* = 0.661 (± 0.031) *X* + 0. (± 0.029) (*r* = 0.952; *N* = 178; *P* < 0.00001). (**B**) Log_10_ resting metabolic rate in relation to log_10_ dry body mass (data for Petersburg, Kanesatake, Ell, Blue and Williamsburg populations from [13]). The regression equations are (*NF*): *Y* = 0.760 (± 0.080) *X* − 4.028 (± 0.252) (*r* = 0.809; *N* = 190; *P* < 0.00001) and (*F*): *Y* = 0.591 (± 0.060) *X* − 3.511 (± 0.186) (*r* = 0.725; *N* = 336; *P* < 0.00001). (C) Log_10_ gill surface area in relation to log_10_ dry body mass (data for Petersburg, Kanesatake, Big Rock, Ell, Blue and Williamsburg populations from [70]). The regression equations are (*NF*): *Y* = 0.743 (± 0.046) *X* + 0.823 (± 0.019) (*r* = 0.962; *N* = 85; *P* < 0.00001) and (*F*): *Y* = 0.620 (± 0.084) *X* + 0.751 (± 0.025) (*r* = 0.829; *N* = 100; *P* < 0.00001).

The scaling exponents for growth rate, resting metabolic rate (*RMR*), and gill surface area for oxygen uptake are all significantly lower in populations from springs with versus without fish (Figure 6). Furthermore, the scaling slopes for these traits are similar within each predation regime, thus indicating that fish predators have caused them to show congruent allometric shifts. In addition, for growth rate and *RMR* the regressions for populations from springs with and without fish intersect at small to medium sizes. As a result, small juveniles tend to have higher rates of growth and metabolism in springs with versus without fish, whereas the opposite occurs for large adults. This size-dependent reversal is not evident for gill surface area because this trait could not be reliably estimated in very small juveniles (see [70]). 

**Figure 7:**
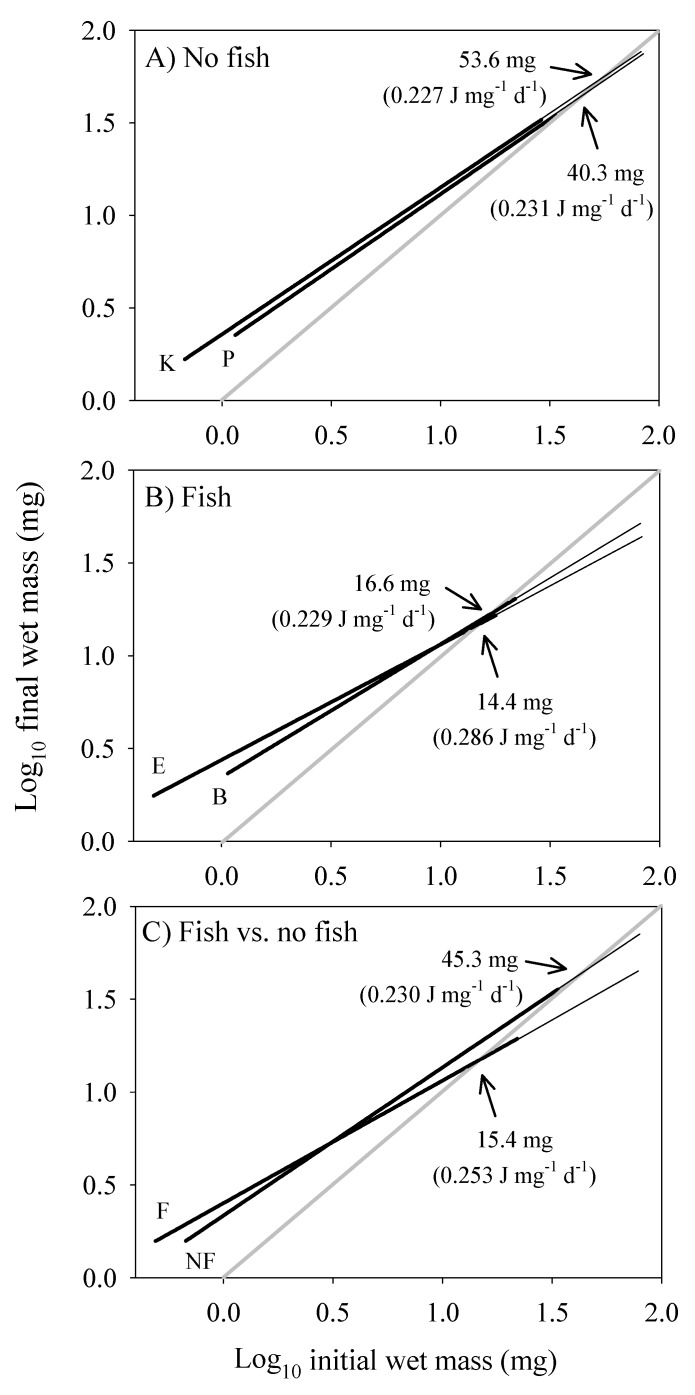
Estimation of the mass-specific cost of body maintenance (J mg^−1^ d^−1^, in parentheses), which is based on the resting metabolic rate at the wet body mass (mg, as shown) at which growth ceases (as indicated by the gray line), as derived from three sets of empirical scaling relationships: final wet mass versus initial wet body mass after a 3 wk culture period (solid black lines), resting metabolic rate versus dry body mass, and dry body mass versus wet body mass for four populations of the amphipod *Gammarus minus* ([13]; and Appendix B). The methods used are described more fully in Section 2.8. The thin black lines represent extrapolations of the growth scaling relationships beyond the body-mass ranges sampled. Shown are comparisons of data for amphipods from the springs without fish predators (P = Petersburg; K = Kanesatake) (**A**), from the springs with fish predators (E = Ell; B = Blue) (**B**), and the collective data from the pairs of springs with versus without fish predators (**C**). Note that the estimated maximal body mass is significantly smaller in the springs with versus without fish, as is observed. The 95% confidence intervals for maximal body mass estimates, calculated using the 95% CI for the growth scaling slopes, are: K (46.8–61.1), P (33.0–49.4), E (13.0–16.0), B (14.4–19.1), F (14.1–16.7) and NF (40.5–50.8).

The scaling of the cost of non-growth body maintenance (*C_M_*) was estimated by determining the *RMR* at the dry body mass at which growth ceased, and extrapolating from that point using a slope = 1 (assuming that the scaling of *C_M_* is volume-related), as shown in Figure 7 (see also Section 2.8). The intersection of the *C_M_* and growth scaling relationships provides an estimate of maximal body mass, which is smaller in populations from springs with versus without fish (Figure 7A–C), as is observed [13]. 

**Figure 8:**
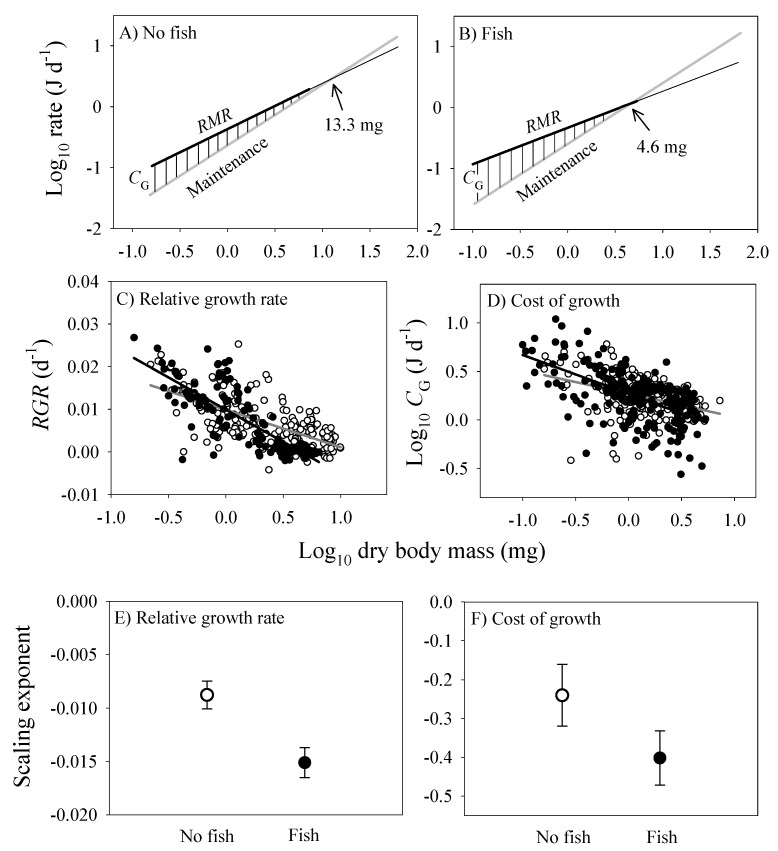
Scaling of log_10_ relative growth rate and log_10_ energy cost of growth with log_10_ dry body mass in the amphipod *Gammarus minus*. The scaling relationships were calculated from data for each of two pairs of populations inhabiting springs without (**A**,**C**,**E**) versus with (**B**,**D**,**F**) fish predators. In (**A**,**B**) the scaling of the cost of growth (*C_G_*) is shown as the hatched area between the scaling relationship for resting metabolic rate (*RMR* = black line) (taken from Figure 6) and that for maintenance energy expenditure (*C_M_*) (gray line) (taken from Figure 7). The dry body mass at which these scaling relationships converge, and at which growth was estimated to stop based on actual measurements, is also shown in each panel. The thin black lines represent extrapolations of the *RMR* scaling relationships beyond the body-mass ranges sampled. In (**C**), the scaling relationships between relative growth rate (*RGR*) and dry body mass are shown for amphipods from springs with (black line and black circles for individual points) versus without fish predators (gray line and open circles). The regression equations (including 95% confidence intervals for each parameter) are respectively *Y* = 0.00991 (± 0.00070) − 0.0151 (± 0.00140) *X* (*r* = 0.849; *N* = 178; *P* < 0.00001) and *Y* = 0.00988 (± 0.00074) − 0.00877 (± 0.00131) *X* (*r* = 0.660; *N* = 228; *P* < 0.00001). In **D**, the scaling relationships between *C_G_* and dry body mass are shown for amphipods from springs with versus without fish predators (indicated as in **C**). The regression equations are respectively *Y* = 0.268 (± 0.0276) − 0.402 (± 0.0698) *X* (*r* = 0.600; *N* = 230; *P* < 0.00001) and *Y* = 0.270 (± 0.0287) − 0.240 (± 0.0795) *X* (*r* = 0.398; *N* = 190; *P* < 0.00001). In (**E**,**F**), the scaling exponents (slopes in the log-log plots shown in **C** and **D**; ± 95% confidence intervals) for *RGR* and *C_G_* are compared between amphipods from springs with (black circles) versus without fish predators (open circles). Note that in both cases the scaling slope is significantly more negative for amphipods from the springs with versus without fish.

The energy cost of growth (*C_G_*) and its scaling with dry body mass were estimated according to equation 6, and by comparing the scaling relationships of *C_M_* and *RMR*. *C_G_* steadily decreases during ontogeny until it reaches zero at the point that growth ceases (Figure 8). This point occurs at a smaller body mass in the populations from springs with versus without fish (compare Figure 8A,B). Furthermore, the relative growth rate (*RGR*) and *C_G_* both decline more rapidly with increasing body mass in the populations from springs with versus without fish (Figure 8C,D). Accordingly, the scaling slopes for *RGR* and *C_G_* are significantly lower in the populations from springs with versus without fish (Figure 8E,F). 

### 3.5. Scaling of Cost of Reproduction of Field-Collected Brooding Females 

Reproductive investment is estimated as total dry mass and number of eggs per brood, which both show significantly higher scaling exponents with dry body mass in populations from springs with fish predators (scaling exponents = 1.184 and 1.034, respectively) than in populations from springs without fish (0.860 and 0.758, respectively) (Table 2; data from [71]). The scaling exponent for mean individual dry egg mass was also higher in populations from springs with versus without fish, but not significantly so (Table 2).

### 3.6. Synthetic Allometry of Energy Intake and Use 

The scaling exponents for various energetically significant traits analyzed in this study are summarized in Table 2. A major pattern that emerges is that most traits related to somatic investment exhibit significantly lower scaling exponents in populations from springs with fish than in populations from springs without fish. In contrast, traits related to reproductive investment exhibit higher scaling exponents in populations from springs with fish than in those from springs without fish. The parallel scaling of energy intake, oxygen-uptake capacity, and rates of growth and metabolism in populations from each predation regime is especially striking. 

## 4. Discussion

Our discussion focuses on five major questions. First, how do the various ontogenetic body-mass scaling exponents that we have observed match with theoretical expectations, such as the surface law and the 3/4-power law that are based on physical and geometric constraints? Second, what roles do bottom-up (resource supply) and top-down (predation) effects likely play in these scaling relationships? Third, do fish predators cause allometric responses in amphipod prey that are congruent (symmorphic), discordant, or a mixture of the two? Fourth, what are the likely causal mechanisms underlying the pervasive effects that fish predators have on the ontogenetic scaling of energy intake and use in amphipods? Fifth, what general implications do our results have for understanding the mechanisms controlling energy expenditure in organisms, especially in reference to various theoretical growth models and other energy-based theories of biological and ecological processes.

### 4.1. Relevance to Physical and Geometric Models of Biological Scaling

Biological scaling relationships often follow power functions with exponents near 2/3 or 3/4. Surface area to volume constraints on resource uptake or metabolic waste removal may yield 2/3-power scaling relationships (‘surface law’ [27,105,106]), whereas size-dependent constraints of anatomical resource-supply networks may yield 3/4-power scaling relationships (3/4-power law [106,107,108]. Our results provide only limited support for these ‘laws’. 

The near 2/3-power scaling of food assimilation rate in the laboratory for *G. minus* populations from springs with and without fish (Figure 1) supports the surface law. Under ad libitum food conditions, gut surface area may be the major limiting factor for food assimilation into the body proper, and since gut surface area scales with exponents often near 2/3, so should assimilation rate (see also [96,97,109,110,111]. In addition, the scaling exponents for laboratory assimilation rate are not significantly different from 3/4 (Table 2), and thus do not contradict the 3/4-power law and resource-supply network models proposed to explain it (e.g., [98,107,112]). Furthermore, the mean scaling exponent (± 95% confidence intervals) for resource consumption rate in a sample of 48 animal species is 0.780 ± 0.052, which is significantly different from 2/3, but not 3/4 (data from [111,113,114,115]). 

However, physical models of biological scaling ignore behavioral and ecological effects. For example, under ad libitum food conditions in the laboratory, fat-poor *G. minus* from springs with fish predators eat more than do fatter amphipods from springs without fish predators. A difference in ‘hunger level’ may have resulted in the observed significant differences in the elevations of the scaling relationships between populations from springs with versus without fish (Figure 1). Inverse correlations between assimilation rate and fat mass among the sampled individuals and populations of *G. minus* support this hypothesis (Figure 2). Also consistent with this hypothesis are the tighter relationships between assimilation rate and body mass observed for population samples from springs with versus without fish (Figure 1A–D). These tighter relationships are indicated by higher correlation coefficients (0.742 versus 0.488: Figure 1F) and the significantly lower residual variances shown by the amphipods from springs with fish (*N* = 109) versus without fish (*N* = 105) (*F* = 4.435; *P* < 0.001; also see Figure 2B). Perhaps when given ample food without the threat of predation, the fat-poor amphipods from the fish springs were more compelled to replenish their low energy stores by maximizing their food intake, relative to their body mass, compared to the fat-rich amphipods from the fishless springs, some of which displayed assimilation rates considerably below the best fit line (compare Figure 1A–D). 

In addition, models focused on the 3/4-power law cannot explain why the presence of predators affects the scaling exponents of multiple energetically significant traits in *G. minus* populations. Although the scaling exponents for growth, metabolism and gill surface area in populations from springs without fish predators are not significantly different from 3/4, these exponents are significantly lower than 3/4 in populations from springs with fish (Figure 6, Table 2). However, the predator-induced shifts in the scaling of growth and metabolism (*RMR*) parallel that for gill surface area, thus providing support for surface effects on biological scaling in *G. minus.* Yet, the direction of causation is an open question, as discussed in Section 4.4. 

### 4.2. Bottom-Up (Resource Supply) Versus Top-Down (Predation) Effects 

According to the metabolic theory of ecology (MTE), size-dependent resource-supply limits to metabolizing cells cause the body-mass scaling of metabolic rate, which in turn drives the scaling of other biological and ecological processes [25,26]. However, the MTE cannot explain why the significant differences in the scaling of *RMR* between *G. minus* population samples from springs with versus without fish predators are not paralleled by similar differences in the scaling of food assimilation rate (Table 2). The rates of energy assimilation and metabolism do not appear to be closely linked, at least during the short time laboratory measurements were made. 

However, laboratory measurements of assimilation rate under ad libitum food conditions do not take into account ecological effects that limit access to food in nature. In particular, a major effect of predators is to inhibit the activity and thus foraging rates of prey organisms (e.g., [2,3,116,117,118], including amphipods (e.g., [90,91,92,93,119,120,121]), and thus presumably their ability to accumulate energy stores. This explains why, in our laboratory assimilation study, amphipods collected from springs with fish predators exhibited significantly lower fat contents than those from springs without fish (Figure 2A). As a result, energy-deficient amphipods from springs with fish exhibited higher short-term assimilation rates in the laboratory, as compared to those of amphipods from springs without fish (Figure 2A). 

In addition, amphipods freshly collected from springs with fish predators exhibited significantly lower fat-mass scaling exponents than those from springs without fish (Figure 3 and Figure 4). This scaling difference appears to be mainly due to the significantly lower fat content of adult amphipods from springs with versus without fish (Figure 5). This difference can be explained as the result of size-selective, visually hunting fish predators causing greater inhibition of foraging activity (and thus accumulation of energy stores) in large conspicuous adults versus small inconspicuous juveniles, (see [20,122]). In contrast, juveniles from populations inhabiting springs with fish exhibited similar or even higher relative fat stores than those from springs without fish. This finding may be explained in part by realizing that adult amphipods are cannibalistic and will feed on smaller individuals, if given the opportunity (reviewed in [123]). In the absence of fish predators, a relatively higher abundance of cannibalistic adults may inhibit the foraging activity of juveniles, thus causing them to have lower fat contents than those in springs with fish. All in all, fat content serves as a useful indicator of accessible food supply, and as such can be used to adjust the scaling exponents of assimilation rate observed under ad libitum food conditions in the laboratory to reflect more realistic conditions in nature. When this is done, the inferred scaling exponents for field assimilation rate closely approximate that for the rates of growth and metabolism (Table 2). 

In short, our results cannot be fully understood unless both bottom-up (resource supply) and top-down (predation) effects are considered. The scaling of energy assimilation, metabolism and growth depend strongly on top-down effects of predators, which in part act via effects on accessible food supply (see also Section 4.4). These supply limits are ecologically caused, and are not simply the result of size-dependent constraints of anatomical resource-supply networks. 

### 4.3. Degree of Congruence of Energetic Allometric Responses by Amphipods to Fish Predators 

The MTE predicts strong congruence among the scaling relationships of diverse biological and ecological processes, all of which should obey the 3/4-power law [25,26]. This kind of congruence is approximated by the amphipods from springs without fish, where the scaling exponents for rates of growth, metabolism and field assimilation rate are all similar to 3/4, though not always significantly so (0.76–0.80: Table 2; see also Section 4.1). However, unexplained by the MTE is the strong similarity of the scaling exponents for these same traits by amphipods from springs with fish that have values significantly lower than 3/4 (0.59–0.66: Table 2). This congruence of scaling relationships for energy intake and expendure can be called ‘symmorphic allometry’. ‘Symmorphosis’ refers to an adaptive matching of the magnitude of structures or processes related to resource supply and demand [124,125]. The idea is that natural selection should favor such matching so that demand does not exceed supply, and vice versa, thus minimizing fitness-reducing resource deficits or excesses. 

Remarkably, fish predators have caused a coordinated shift in the scaling of several processes related to somatic maintenance, including energy assimilation, metabolism, growth and oxygen-uptake capacity (as indicated by gill surface area). In addition, fish predators have caused a coordinated shift in the scaling of traits related to reproductive investment, but in the opposite direction. In particular, total mass and number of eggs per brood show significantly higher scaling exponents in populations from springs with versus without fish (1.03–1.18 vs. 0.76–0.86), in contrast to the decrease in scaling exponents shown for traits involving somatic investment (Table 2). These opposing allometric shifts have led to an intensified trade-off between somatic and reproductive investments in amphipods exposed to fish predators. When the risk of predation is high, amphipods show earlier and greater ontogenetic shifts in their energy allocation toward reproduction and away from somatic maintenance and growth. This response is consistent with life-history theory that predicts that organisms subject to size-selective predation should mature and invest more in reproduction at an earlier age, thereby allowing them to perpetuate their genes before they are eaten, likely at a relatively young age [19,126,127,128,129]. 

### 4.4. Mechanisms Underlying Effects of Fish Predators on the Energetic Allometry of Amphipod Prey 

**Figure 9:**
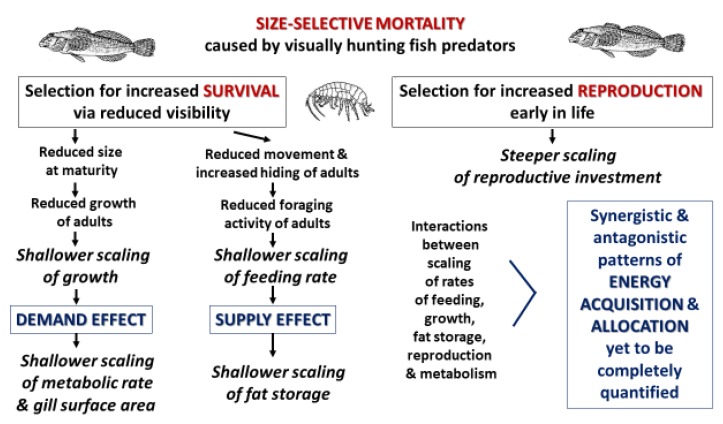
Flow diagram depicting hypothetical mechanisms underlying effects of size-selective predation by visually hunting fish on the ontogenetic body-mass scaling of various energetically significant traits related to somatic and reproductive investment in the freshwater amphipod *Gammarus minus*. This diagram features adaptive evolutionary responses to size-specific selection for increased adult survival via reduced visibility and increased reproduction early in life. Interactions among the various scaling relationships for energy intake and expenditure have yet to be fully quantified and understood.

Our current tentative view of the mechanisms by which fish predators have caused shifts in the energetic allometry of *G. minus* is outlined in Figure 9. This hypothetical picture focuses on how size-selective predation by visually hunting fish predators has caused adaptive evolutionary responses in *G. minus* populations that increase adult survival and reproduction. Shallower symmorphic scaling of various energetically significant traits related to somatic investment can be explained in terms of selection for reduced size at maturation and reduced behavioral activity of adults, both of which reduce ‘visibility’ to lurking fish predators. In addition, steeper scaling of reproductive investment may be the result of selection for greater reproduction at earlier ages and smaller sizes so as to increase the likelihood of offspring production before death by predation. Our conceptual view highlights effects of both resource supply and demand, but their relative roles and possible interactive effects in causing the scaling relationships that we have observed are yet to be conclusively and comprehensively determined.

Accessible resource supply appears to be importantly involved in the ontogenetic body-mass scaling of fat mass in *G. minus* populations. Reduced foraging activity by large conspicuous adults in springs with fish predators likely reduces their food intake and accumulation of fat stores (Figure 5), thus causing a shallower scaling of fat mass, as compared to that of populations in springs without fish (Figure 3 and Figure 4). Limited food supply resulting from reduced foraging may also favor a more conservative energetic lifestyle, including reduced growth and metabolism, in adults inhabiting springs with versus without fish predators (following the hypothesis of [48]; also see [130]). If so, reduced accessible resource supply may also play a role in the shallower scaling of growth and *RMR* in *G. minus* populations from springs with versus without fish.

However, several observations contradict or complicate a simple resource-supply centered view. First, laboratory measurements show that the scaling of *RMR* and food assimilation rate are not closely linked. The scaling exponents of *RMR* differ significantly between populations from springs with versus without fish, whereas the scaling exponents for assimilation rate do not (Table 2). A similar mismatch between the scaling of *RMR* and assimilation rate has been reported for the tobacco hornworm *Manduca sexta* [131]. Second, differences in *RMR* scaling were observed in the absence of direct predation risk and in the presence of ad libitum food until 24 h before respiration measurements were taken (see also [13]). Third, in the presence of fish predators, the effect of reduced accessible food supply on fat storage in adult amphipods should result in changes in tissue resource demand that may confound a simple effect of resource supply on *RMR*. Reduced amounts of metabolically inert fat tissue should cause an increase in mass-specific *RMR*, not a decrease as predicted by a supply-centered view. These countervailing effects require further study. Fourth, amphipods from springs without fish did not show altered patterns of growth (as measured in field enclosures) when transplanted into springs with fish (see [13]). This result suggests that growth patterns in *G. minus* are the result of long-term genotypic adaptation, rather than being phenotypically plastic responses to the presence of intimidating fish. Fifth, a supply-centered view cannot explain why adult females of *G. minus* expend energy for reproduction at smaller sizes and show steeper ontogenetic scaling of reproductive investment, despite reduced energy intake in springs with versus without fish predators (see also [71]). 

The above observations suggest that effects of fish predators on accessible resource supply cannot completely explain the shifts in energetic allometry of *G. minus* that have been observed. Effects on resource demand, including by growth, locomotion and reproduction, should also be considered. Many studies have shown that growth and locomotor activity have major effects on metabolic scaling (reviewed by [27,28,100,132,133]). Furthermore, predators can affect both of these resource-demanding processes, as commonly observed (e.g., [1,2,3,23,36,42,43,44,48,116,117,118,134,135]), including in amphipods (e.g., [8,91,121,122,130,136,137,138,139,140]). 

Life-history theory predicts that higher mortality rates in large adults versus small juveniles selects for maturation at smaller sizes and minimal postmaturational growth [127,128,129], as occurs in *G. minus* populations in springs with size-selective fish predators [13]. Remarkably, amphipods in springs with fish exhibit determinate growth (little or no postmaturational growth), whereas in springs without fish they display indeterminate growth (continuing postmaturational growth). Since growth is energetically expensive, its demand for resources has a large impact on *RMR*, especially in ectothermic amphipods with low maintenance requirements. Thus, the predator-induced shift in the ontogenetic scaling of growth is paralleled closely by the scaling of supporting metabolism and oxygen-uptake capacity (as indicated by gill surface area) (Figure 6). 

Effects of inter-population differences in size-specific growth on metabolic scaling in *G. minus* have been interpreted as being the result of life-history evolution mediated by size-selective predation [13]. However, predators or their cues may also have direct physiological effects on the growth and maturation of prey. For example, fish-predator cues cause reduced size at maturation in the cladoceran *Daphnia magna* by physiological effects on the relative rates of growth and maturation, that are not simply the result of resource-supply effects resulting from reduced foraging activity [37]. Both food supply and direct physiological effects of predator cues should be considered because they can have interactive effects on growth rate (e.g., [38]). 

When exposed to fish predators, relatively large adult amphipods also reduce their locomotor activity (e.g., [20,122]). After multiple generations, natural selection may favor less athletic bodies with reduced metabolic machinery in amphipods from springs with versus without fish. If so, decreases in routine locomotor activity (in addition to reduced growth) could help explain why adult amphipods in springs with fish tend to have lower *RMR*s than those in springs without fish, hence resulting in a shallower scaling of *RMR*. This hypothesis is currently being tested. 

As argued by [30], both resource supply and demand can affect metabolic scaling. Both should be considered when assessing effects of predators on the energetic allometry of prey organisms. Resource-supply centered models (e.g., [25,107,112]) offer incomplete explanations of biological scaling. Resource-demanding processes may also have major effects [13,27,28,29,30]. Furthermore, although the MTE assumes that resource supply limits metabolic rate, which in turn drives the rates of other biological and ecological processes, it is also possible that various resource-demanding processes drive metabolic rate, or that multiple processes related to energy intake and use have coevolved in a symmorphic way (cf. [13,28]), as we have observed in our study (Figure 6; Table 2). An increased focus on how resource-demanding processes affect metabolic scaling is especially needed because of growing evidence contradicting the view that size-specific limits on internal resource supply to metabolizing cells cause metabolic scaling (e.g., [29,30,141,142,143,144]. 

### 4.5. Implications for Growth Models and Other Energy-Based Biological and Ecological Theories 

In this section we discuss further theoretical implications of our work. First, we make brief remarks regarding the implications of our findings for specific features of three major growth models based on energetic scaling relationships. Growth is of essential importance because it is the process that produces the array of body sizes analyzed in ontogenetic body-mass scaling studies. Second, we discuss general implications of our findings for energy-based biological and ecological theories. 

#### 4.5.1. Specific Implications for Growth Models Based on Energetic Scaling Relationships

**Figure 10:**
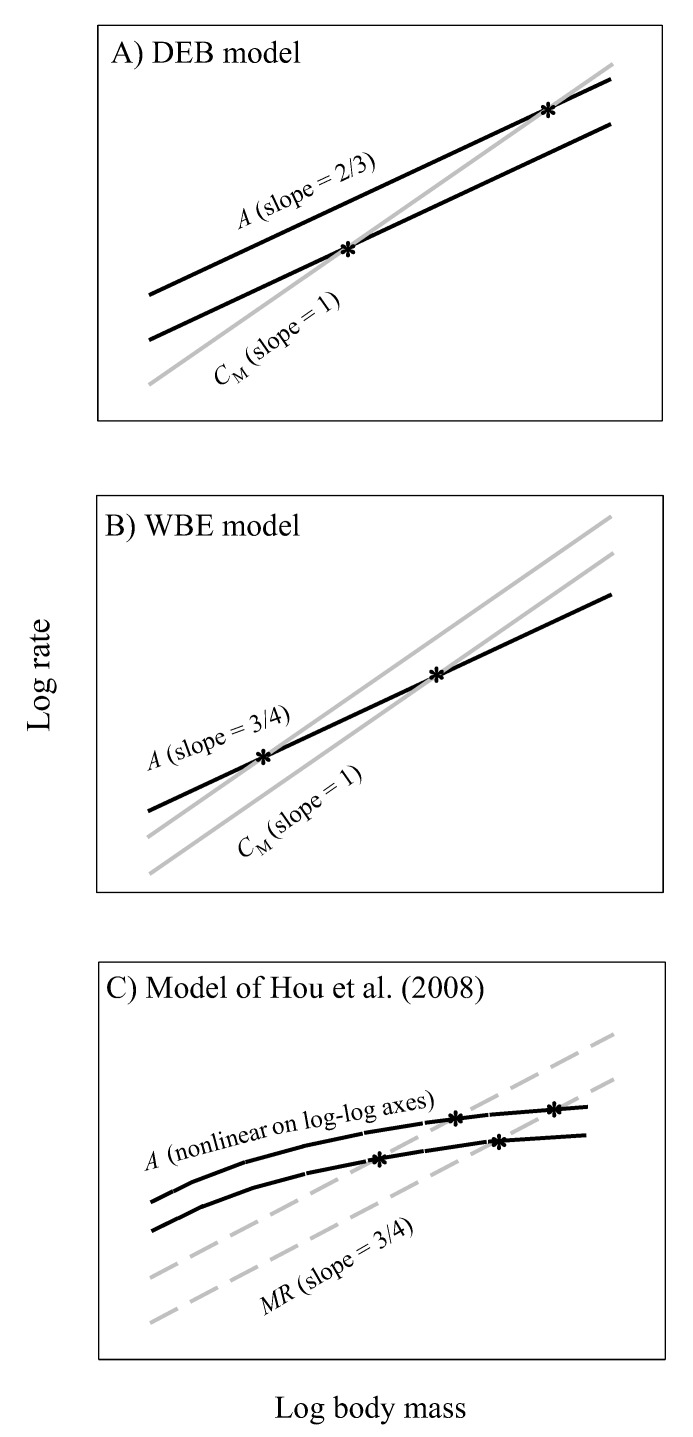
Schematic diagrams illustrating predictions of maximal adult body mass or volume (indicated by black dots) based on three growth models. In (**A**,**B**), the maximal body mass or volume is predicted to occur at the intersection of the scaling relationships for the rates of food-energy assimilation (*A*, red lines) and energy expenditure for body maintenance (C_M_ or C_V_ scaled to body mass and structural volume, respectively, as indicated by blue lines). Different maximal body masses are predicted based on variation in the elevation of the scaling relationship for *A* (following the DEB model of [96,97]) or *C_M_* (following the WBE model of [98]). The DEB and WBE models also differ in positing that *A* should scale to the 2/3-versus 3/4-power, respectively. In (**C**), the maximal body mass is predicted to occur at the intersection of the scaling relationships for *A* and total metabolic rate (*MR*, green lines). According to the model of [147], *A* scales curvilinearly with body mass, whereas *MR* scales linearly to the 3/4-power. Different maximal body masses are predicted based on variation in the elevation of the scaling relationships for either *A* or *MR*. DEB: dynamic energy budget theory; WBE: West, Brown and Enquist.

Energy-based growth models typically compare the ontogenetic scaling relationships for energy intake and body maintenance energy costs to predict growth curves and maximal body size. Energy available for growth is assumed to equal energy intake minus energy used for maintenance. When this difference becomes zero, growth ceases. These kinds of models have a long history (e.g., [145,146]), but here we focus on three recent notable models, the (DEB) model based on dynamic energy budget theory [96,97], the (WBE) model of West, Brown and Enquist based on resource-supply network theory [98], and the Hou et al. model, which is a modification of the WBE model [147] (Figure 10). Although the DEB and WBE models both assume that maintenance costs scale to the 1 power, the DEB model assumes that assimilation rate (*A*) scales to the 2/3-power (Figure 10A), whereas the WBE model assumes that the exponent is 3/4 (Figure 10B). In contrast, the Hou et al. model assumes that *A* scales curvilinearly (concave downward) in log-log space, which is compared to the assumed 3/4-power scaling of ‘total metabolic rate’ (*MR*, including *RMR* and costs of feeding and routine activity) to predict growth curves (Figure 10C). 

Our results both support and contradict specific aspects of these models. First, the near 2/3-power scaling of assimilation rate by *G. minus* in the laboratory (Figure 1F) best matches that assumed by the DEB model (assuming that dry body mass used to estimate body size in our study is proportional to structural volume, which is used to estimate body size in the DEB model; but see below). However, the scaling exponents that we observed for various populations are also not significantly different from 3/4 (Figure 1), as assumed by the WBE model. Our assumption that maintenance costs scale isometrically is also consistent with these models (but see Section 4.5.2). However, although the WBE model predicts that smaller maximal body sizes (as occur in *G. minus* populations from springs with fish predators) should result from higher maintenance costs, the mass-specific cost of maintenance (C_M_) was calculated to be only slightly higher for populations from springs with versus without fish (253 versus 230 J mg^−1^ d^−1^; Figure 7C), and this larger estimate is due to a relatively large value for only one of the populations in the springs with fish (0.286 for Blue Spring amphipods, compared to 0.227 to 0.231 for the other three populations: see Figure 7A,B). 

Contrary to the Hou et al. model, assimilation rate scales log-linearly in all four population samples of *G. minus* (Figure 1). In no case, does a curvilinear polynomial (quadratic) regression fit the data significantly better than a linear regression (the quadratic term is never significant: *P* > 0.20). In addition, the Hou et al. model uses *MR* as a measure of non-growth costs for maintenance and routine activity, but this is problematic because this measure of metabolic rate includes the metabolic cost of growth (Figure 8A,B). Therefore, it cannot be used as an independent measure to calculate the amount of surplus energy available for growth (cf. [148]). For this purpose, assimilation rate should be compared with non-growth maintenance costs, as assumed by the DEB and WBE models. In addition, the Hou et al. model assumes that *RMR* and *MR* scale with an exponent of 3/4, but in *G. minus* populations from springs with fish predators, the *RMR* scaling exponent is significantly less than 3/4. Furthermore, given that the Hou et al. model assumes that the scaling exponents for *MR* and *RMR* should be the same, it is not able to predict the maximal body masses actually observed in *G. minus* populations because of a lack of intersection between the parallel scaling relationships for *RMR* (and thus presumably *MR*) and assimilation rate. In *G. minus* populations from springs with or without fish predators the scaling exponents for *RMR* are not significantly different from those inferred for assimilation rate in the field (see Table 2). 

Moreover, all of these models ignore ecological effects on the scaling of assimilation rate. Our calculations suggest that the scaling exponents for assimilation rate are different in the field than in the laboratory (Table 2), and not fixed at 2/3 or 3/4. The inferred scaling exponent for field assimilation rate is lower in *G. minus* populations from springs with versus without fish predators (Table 2). As a result, predicted maximal body masses should be lower in the populations from springs with versus without fish (as inferred from comparing the exponents of 0.623 and 0.796 for field assimilation rate, respectively, versus 1 for maintenance), as observed (Figure 7 and Figure 8A,B). This prediction relies on differences in the assimilation scaling exponent, and not elevation, as assumed by the DEB and Hou et al. models (Figure 10A,C). Therefore, we recommend that future tests of growth models should use energetic scaling relationships obtained from data collected in nature, not in the laboratory, and that they should consider variation in both slopes and elevations of these relationships. 

DEB theory is quite flexible, and could be adjusted to include ecological effects in two possible ways by using empirical data. First, the mathematical component that specifies effects of food availability on assimilation rate [97] could be modified to include size-related and size-independent changes, thus altering either or both the scaling exponent and elevation for assimilation rate with body size. Second, changes in metabolically inert fat content that accompany variation in food availability (see Section 3.3) could be used to modify the scaling of maintenance costs in relation to body mass. By scaling assimilation rate and maintenance costs against structural volume (Figure 10A), conventional DEB theory ignores possible effects of reserve materials (e.g., fat), which are considered a separate body component [96,97], on these energetic processes. However, including the effects of food availability on size-specific assimilation rate, and of body reserves on size-specific maintenance costs, may allow DEB models to account for the effects of fish predators on the mass-related energetics of amphipods that we have observed, a possibility worth examining. 

#### 4.5.2. General Implications for Energy-Based Biological and Ecological Theories 

Our results show the importance of not only considering ecological effects on biological scaling relationships, but also the role that biological regulation plays in mediating these effects. The growth models considered in Section 4.5.1 all assume that growth curves are the passive result of the ontogenetic scaling of energy intake in relation to that for maintenance costs, both of which are dictated by physical and geometric constraints. These models assume that the scaling of assimilation rate is dictated by resource-uptake surface area based on Euclidean or fractal geometry, whereas maintenance costs are dictated by the volume of metabolizing tissue (see also Section 4.1). However, the scaling of assimilation rate may also be affected by ecological factors, such as size-biased predation risk, thus causing deviations in scaling exponents from the theoretical values of 2/3 or 3/4. In addition, the scaling of maintenance costs may depend on ontogenetic changes in the relative resource demand of various biological processes (e.g., thermoregulation) and the relative proportions of tissues with high versus low metabolic needs [27,100,132], thus causing deviations in scaling exponents from the theoretical value of 1. Accounting for these effects and their mediation by biological regulation is challenging, but would make growth models more realistic.

Growth is not a simple passive result of fixed, physically constrained energetic scaling relationships, but also depends on the active, flexible regulation of various energetic processes. It is also not the simple result of the driving influence of resource-supply limited metabolic rate, as claimed by the MTE. The importance of adaptive optimization, as mediated by biological regulation, is seen in how malleable the scaling exponents are for various energetic processes in populations of *G. minus*, shifting markedly in response to predation risk. It is also seen in how growth and metabolism have reciprocating effects that lead to their co-adjustment by various regulatory factors (reviewed in [28]). We suggest that what is driving growth rate is not size-specific metabolic rate, but size-specific mortality rate. This view helps to explain why the scaling of traits for somatic investment responds differently to predation risk than does the scaling of traits for reproductive investment. Increased mortality on adults relative to juveniles favors a decreased resource-allocation priority on parents of the present generation versus an increased priority on offspring of the future generation. An adaptive regulatory view also helps explain why size-specific predation risk can cause various energetic processes related to somatic investment to decrease in large adults, but increase or stay the same in small juveniles (see the crossing scaling relationships for populations from springs with versus without fish predators: Figure 2, Figure 3, Figure 6 and Figure 8C,D). These divergent age-and size-specific responses to predation risk may help explain why studies in the literature based on specific life stages have shown both positive and negative responses of metabolic rate by prey to the presence of predators or their cues (Table 1). This variation in metabolic responses may also relate to different anti-predator strategies and other biological, ecological and methodological factors discussed further in Appendix C. As such, this physiological variation also highlights the ecological sensitivity and actively regulated malleability of energetic processes in organisms. 

Organisms can be considered “informed resource users” [149]. The acquisition and use of both energy and information are essential to life and its evolutionary success. Therefore theoretical models of various biological and ecological processes should be based on not only energy, as are the MTE and DEB theory, but also information transmitted by various biological regulatory systems [28]. 

## 5. Conclusions

Major general conclusions and recommendations of our study include:

1. To fully understand the effects of predators on their prey, it is important to consider all of the life stages of prey organisms. Ontogenetic body-mass scaling analyses, as carried out in our study, are one useful way to do this.
2. Given the importance of energy for all biological processes, an examination of how predators affect the ontogeny of various energetically significant traits, and their scaling with body mass, can provide valuable insights into age- and size-specific energy-allocation strategies (also see [22,130,150,151]).
3. Various biological processes, such as energy intake and use for various vital functions (e.g., metabolism, growth, locomotion and reproduction) are interdependent in synergistic or antagonistic ways, and thus should be examined with an integrated, holistic perspective. By doing so, our study of the freshwater amphipod *Gammarus minus* has revealed the ‘symmorphic allometry’ (parallel scaling) of two different clusters of energetically significant traits based on somatic versus reproductive investments that have shifted in coordinated ways in response to the presence of fish predators. With regard to somatic investment, predation risk has caused shallower, congruent scaling of the rates of assimilation, growth and metabolism, fat content, and oxygen uptake capacity (as indicated by gill surface area). Accordingly, adults are smaller and leaner, and have slower rates of growth and metabolism and smaller gills, relative to their body mass, in springs with versus without fish predators. In contrast, traits related to reproductive investment (e.g., total mass and number of egg per brood) scale more steeply in springs with versus without fish predators. Consequently, predation risk intensifies the trade-off between somatic and reproductive investments in *G. minus*.
4. Our results add to growing evidence that biological scaling is ecologically sensitive and evolutionarily malleable, and not merely physically constrained by body design (e.g., [27,29,132,144,152,153,154,155,156,157]). They also show that biological scaling can be affected by both top-down (predation) and bottom-up (resource supply) factors.
5. Our findings support the view that growth is not merely the passive result of the body-mass scaling of assimilation rate and maintenance costs, as determined by physical constraints, but is actively regulated to maximize evolutionary fitness in local environments [28]. A comprehensive understanding of biological scaling and other aspects of living systems should be based on not only energy, but also information (also see [28,149,158]).
6. Our study of the scaling of various energetically significant traits related to somatic investment focuses on juveniles and adult males to avoid complications regarding egg production. Future research should also examine adult females, because their metabolic responses to predation risk may differ from that of males (see e.g., [22]). In the presence of predators, adult females of *G. minus* invest more in egg production earlier in life and at smaller body sizes, which may draw energy away from somatic growth and fat storage (cf. [130]). This may, in part, explain why females are smaller than males (in addition to sexual selection for larger size in males to increase their mating success [159,160,161,162]). How the different reproductive strategies of females and males affects their responses to fish predators should be explored.
7. We have presented preliminary results on a complex issue. To clarify further our understanding of anti-predator prey responses, we recommend additional research objectives. First, the scaling of key energetic processes, such as food consumption, metabolism and locomotor activity, should be estimated in nature and not just in the laboratory (see e.g., [65]). Second, interactions between various anti-predator responses by prey (e.g., changes in their rates of feeding, metabolism, growth, reproduction and behavioral activity: see e.g., [13,23,24,38,44,45,48,49,130,163]), and reciprocal effects of these responses on the vulnerability of prey to predation should be investigated (following [24,61,164]). Third, effects of other environmental factors (e.g., temperature, habitat, parasites, and food quantity and quality) on prey responses to predator risk deserve further attention (see e.g., [6,38,43,58,116,119,120,165,166,167,168,169,170,171,172,173,174,175,176,177]). Fourth, the relative roles of evolutionary adaptation and phenotypically plastic acclimation involved in prey responses to predators require elucidation (see e.g., [13,48,166,168,178,179,180]).

## Figures and Tables

**Table 2:** Scaling exponents (slopes) ^1^ of various energetically significant traits in populations of *Gammarus minus* from springs with versus without fish predators ^2^.

Trait	Scaling Exponent for Fish Spring Populations	Scaling Exponent for Non-Fish Spring Populations
**Somatic investment**		
Lab assimilation rate ^3^	0.671 (±0.117; 109)	0.682 (±0.239; 105)
Fat mass (juveniles & adult males) ^4^	**0.952** (±0.091; 76)	**1.114** (±0.059; 75)
Fat mass (juveniles & adult non-brooding females) ^4^	**1.146** (±0.131; 77)	**1.337** (±0.072; 75)
Field assimilation rate ^5^	*0.623*	*0.796*
Growth rate	**0.661** (±0.031; 178)	**0.798** (±0.030; 228)
Resting metabolic rate	**0.591** (±0.060; 336)	**0.760** (±0.080; 190)
Gill surface area	**0.620** (±0.084; 100)	**0.743** (±0.046; 85)
**Reproductive investment**		
Total mass of eggs per brood	**1.184** (±0.211; 83)	**0.860** (±0.232; 114)
Egg number per brood	**1.034** (±0.217; 83)	**0.758** (±0.220; 114)
Individual egg mass	0.151 (±0.096; 83)	0.100 (±0.105; 114)

^1^ The 95% confidence intervals and sample sizes are in parentheses. ^2^ **Bold** numbers indicate scaling exponents that are significantly different between populations from springs with versus without fish. ^3^ Estimated in laboratory. ^4^ Estimated in amphipods collected in field. ^5^ Calculated by adjusting scaling exponents estimated in the laboratory using scaling exponents of fat content (a proxy for food intake) estimated from field-collected juvenile and male amphipods (for details see text).

**Figure A1:**
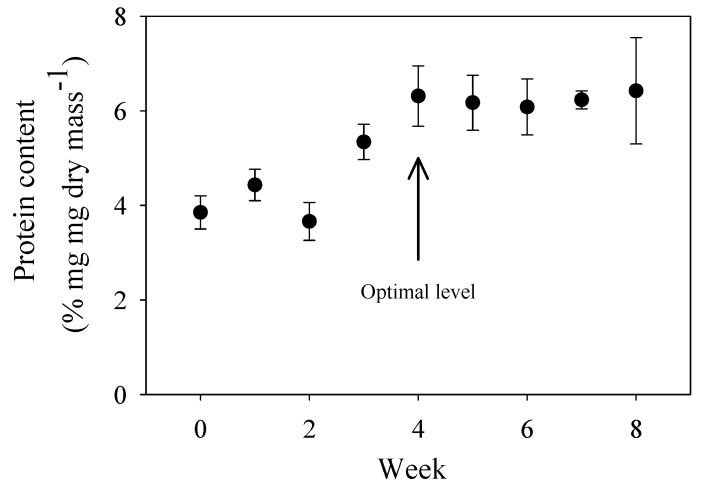
Protein content (mean ± 2 S.E.; *N* = 6–10) of elm (*Ulmus americana*) leaf disks submerged in 0.3-mm mesh bags for various lengths of time in Ell spring during June-August 1992. Protein content was determined by a Bio-Rad (Bradford) protein assay using bovine serum albumin as a standard. Increased protein content is a good indicator of microbial and fungal growth (“conditioning”) because microbes and fungi have a high protein content, whereas dead leaves have a very low protein content. Similar increases in protein (nitrogen) content during detrital leaf conditioning have been observed in other studies (e.g., [181], and other references cited in [86]). The arrow indicates the optimal level of protein content, which represents the highest level achieved over the shortest culture time. Therefore, leaf disks that had been conditioned for 4 weeks were used in the laboratory assimilation study.

## Supplementary Materials

The following are available online at https://www.mdpi.com/2079-7737/9/3/40/s1, Table S1. Log_10_-transformed values of dry body mass (mg), assimilation rate (J/d) and fat mass (mg) of 214 individual amphipods (*Gammarus minus*) collected from four freshwater spring populations. These values were used in the analyses presented in Figure 1 and Figure 2.

## Appendix A

Leaf disks used in the laboratory assimilation study were conditioned in Williamsburg Spring to increase their nutritional quality (see Section 2.3). This conditioning involved the colonization of protein-rich microbes and fungi, which was assessed by a protein assay. The optimal time of conditioning was determined when peak protein levels were reached for leaf samples submerged in a spring with similar water temperature and chemical composition (Ell Spring) for various lengths of time (Figure A1).

## Appendix B

Measurements of wet body mass used in the growth study were converted to dry body mass using the empirical scaling relationships given in Table A1.

**Table A1:** Log_10_ dry body mass (*M_D_*) as a function of log_10_ wet dry mass (*M_W_*) (measured as mg) in four spring-dwelling populations of *Gammarus minus*. Least squares regression equations are shown with 95% confidence intervals for the intercept and slope in parentheses. Associated Pearson’s product-moment correlation coefficients (*r*), sample sizes (*N*) and levels of significance (*P*) are also given. Body masses were measured in amphipods used in the growth study (also see [13]).

Spring Population	Linearregression Equation	Regression Statistics		
		*r*	*N*	*P*
Petersburg	*M_D_* = −0.478 (± 0.037) + 0.957 (± 0.034)*M_W_*	0.988	92	<0.00001
Kanesatake	*M_D_* = −0.502 (± 0.025) + 0.992 (± 0.022)*M_W_*	0.993	116	<0.00001
Ell	*M_D_* = −0.489 (± 0.032) + 0.975 (± 0.034)*M_W_*	0.987	91	<0.00001
Blue	*M_D_* = −0.509 (± 0.048) + 0.977 (± 0.047)*M_W_*	0.983	63	<0.00001

## Appendix C

Predators may cause increases or decreases in the metabolic rate of prey (Table 1). This variation can be explained in several nonexclusive ways. First, some of this variation may relate to age- and size-specific differences in how prey organisms respond metabolically to predators, as seen in our study (also see Section 4.5.2). In *Gammarus minus*, exposure to fish predators (*Cottus cognatus*) that preferentially eat large conspicuous adults results in mass-specific metabolic rate declining in adults, but increasing in small, inconspicuous juveniles (Figure 6B; also see [13]). Adults benefit from slowing their growth and behavioral activity, which in turn requires lower metabolic support. However, juveniles benefit from increased metabolic rates that support rapid growth and activity to ensure maturation and reproduction before they are eaten (also see Section 4.4).

Second, metabolic responses by prey may depend on the duration and magnitude of exposure to predators. Short-term exposure may elicit temporary alarm reactions, mediated by ‘fight or flight’ stress hormones (cf. [23,38]). The immediate motivation may be to flee or seek refuge, thus increasing metabolic rate supporting these escape responses. However, long-term responses may result in a decrease in metabolic rate because of the need to adapt to the energetic consequences of chronic exposure to predators (e.g., increased risk of foraging that reduces food energy intake, and a need to reduce metabolically generated oxidative damage: see e.g., [65,182]). However, the effect of time exposure can be complex, as shown by [35]. In addition, various kinds of prey responses depend on the magnitude of the predation threat [183,184,185], which may also apply specifically to metabolic responses.

Third, variation in metabolic responses may relate to the overall anti-predator strategy of a species. For example, prey species that typically use ‘freezing’ or hiding behavior in response to the approach of a predator tend to exhibit reduced metabolic rates (e.g., [31,32,45,47,62]). In contrast, prey species that use escape behavior tend to exhibit increased metabolic rates that help energize increased activity [33,63].

Fourth, metabolic responses may depend on the type of predator or its cues, and whether the predator is actively approaching or merely in the vicinity [186]. For example, chemical cues indicate that a predator is close, whereas visual cues indicate whether a predator is approaching [187], which may elicit different responses.

Fifth, metabolic responses of prey may differ between aquatic and terrestrial environments because of different modes of predator detection. In water, prey mainly use chemical and tactile cues, whereas on land, they more often use visual and auditory cues (see discussion in [32]).

Sixth, metabolic responses may depend on the vulnerability of prey organisms. Availability of cover or refuges may ameliorate these responses [31].

Seventh, metabolic responses based on short-term phenotypic acclimation of individuals may differ from those occurring via adaptive evolution in populations (see Table 1).

In short, metabolic responses to predation risk likely depend on multiple biological and ecological factors.
